# Clinical trials of disease-modifying agents in pediatric MS: Opportunities, challenges, and recommendations from the IPMSSG

**DOI:** 10.1212/WNL.0000000000007572

**Published:** 2019-05-28

**Authors:** Emmanuelle Waubant, Brenda Banwell, Evangeline Wassmer, Maria-Pia Sormani, Maria-Pia Amato, Rogier Hintzen, Lauren Krupp, Kevin Rostásy, Silvia Tenembaum, Tanuja Chitnis, on behalf of the IPMSSG

**Affiliations:** From the UCSF MS Center (E.W.), San Francisco, CA; The Children's Hospital of Philadelphia (B.B.), Perelman School of Medicine, University of Pennsylvania; Birmingham Children's Hospital (E.W.), UK; Department of Health Sciences (M.-P. S.), University of Genova and Ospedale Policlinico San Martino IRCCS; Department NEUROFARBA (M.-P.A.), University of Florence, Italy; IRCCS Fondazione Don Carlo Gnocchi (M.-P.A.), Florence, Italy; Department of Neurology (R.H.), Erasmus MC, Rotterdam, the Netherlands; MS Comprehensive Care Center at NYU Langone (L.K.), New York, NY; Division of Paediatric Neurology (K.R.), Children's Hospital Datteln, University Witten/Herdecke, Datteln, Germany; Pediatric MS Center (S.T.), Department of Neurology, National Pediatric Hospital Dr. Garrahan, Buenos Aires, Argentina; and Partners Pediatric MS Center (T.C.), Massachusetts General Hospital, Boston.

## Abstract

**Objective:**

The impetus for this consensus discussion was to recommend clinical trial designs that can deliver high-quality data for effective therapies for pediatric patients, in a reasonable timeframe, with a key focus on short- and long-term safety.

**Methods:**

The International Pediatric Multiple Sclerosis Study Group convened a meeting of experts to review the advances in the understanding of pediatric-onset multiple sclerosis (MS) and the advent of clinical trials for this population.

**Results:**

In the last few years, convincing evidence has emerged that the biological processes involved in MS are largely shared across the age span. As such, treatments proven efficacious for the care of adults with MS have a biological rationale for use in pediatric MS given the relapsing-remitting course at onset and high relapse frequency. There are also ethical considerations on conducting clinical trials in this age group including the use of placebo owing to highly active disease. It is imperative to reconsider study design and implementation based on what information is needed. Are studies needed for efficacy or should safety be the primary goal? Further, there have been major recruitment challenges in recently completed and ongoing pediatric MS trials. Phase 3 trials for every newly approved therapy for adult MS in the pediatric MS population are simply not feasible.

**Conclusions:**

A primary goal is to ensure high-quality evidence-based treatment for children and adolescents with MS, which will improve our understanding of the safety of these agents and remove regulatory or insurance-based limitations in access to treatment.

The diagnosis of multiple sclerosis (MS) in children is enabled by diagnostic criteria.^1,2^ Genetic and environmental risk factors contributing to MS susceptibility are shared between pediatric- and adult-onset disease.^3–6^ Pathologic analyses support the same pathologic features of perivenular inflammation, focal demyelinating plaques, and axonal injury.^7^

Pediatric MS follows a relapsing-remitting (RR) course, with a high relapse frequency.^8,9^ MRI features are similar in lesion distribution as adult-onset MS, with the rate of lesion accrual being potentially higher in pediatric patients.^10–12^ Despite frequent relapses and MRI evidence of active disease, pediatric patients with MS rarely accrue significant physical disability in the first decade after disease onset.^13^ In contrast, clear cognitive changes occur even within the first year after disease onset, raising the issue of early treatment intervention.^14,15^

The literature regarding treatment of pediatric MS is largely restricted to retrospective studies of first-line injectable therapies, a few of the oral agents, and natalizumab. While such analyses cannot formally address treatment efficacy, the available data support effectiveness as compared to pretreatment, as well as a similar tolerability and short-term safety profile as has been documented for these agents in adult RRMS trials.^16–26^

Several studies, including a high-profile meta-analysis, support MRI lesions as a valid surrogate endpoint for clinical relapses in RRMS,^27^ opening new possibilities for pediatric trials.

Our goals are to review the current state of pediatric MS clinical trials and lessons learned over the last 6 years and provide consensus recommendations for future trials.

## Current status of clinical trials in pediatric MS

To date, clinical trials of promising MS agents are typically performed in patients 18 years and above. The Food and Drug Administration (FDA), as a consequence of the Pediatric Research Equity Act (congress.gov/108/plaws/publ155/PLAW-108publ155.pdf), and the European Medicines Agency (EMA), as a consequence of the Paediatric Regulation (ema.europa.eu/ema/index.jsp?curl=pages/regulation/document_listing/document_listing_000068.jsp&mid=WC0b01ac0580925c45), mandate pediatric investigation plans to test safety and efficacy of new agents for patients under the age of 18 for drugs approved in adults. As a result, over the last 6 years, 5 clinical trials testing 4 agents have been launched in pediatric MS (table 1).

**Table 1:**
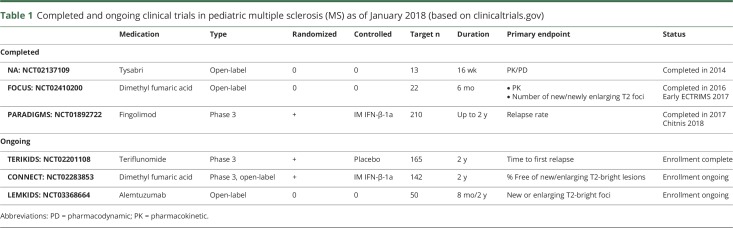
Completed and ongoing clinical trials in pediatric multiple sclerosis (MS) as of January 2018 (based on clinicaltrials.gov)

Strong correlations between treatment effect on relapse rate and MRI lesion accrual have been reported both in adult and pediatric MS.^27–29^ These correlations emphasize the crucial role of MRI in clinical trials of MS agents with anti-inflammatory properties. In particular, a large meta-analysis published in 2013^27^ validated the role of MRI lesions as a surrogate endpoint for clinical relapses in RRMS, confirming previous results^28^ and validating the quantitative relationship that allows estimation of the treatment effect on relapses from an observed effect on MRI lesions.^27^ These findings have strong implications for the design of pediatric trials testing drugs already studied in the adult RRMS population.

## Methods for the updated consensus

The International Pediatric Multiple Sclerosis Study Group (IPMSSG) is a group of international pediatric MS care providers that was established in 2005. The IPMSSG (ipmssg.org/) goals regarding the advancement of clinical care in pediatric MS are listed in table 2. There are currently 165 members registered with the IPMSSG representing 44 countries. The IPMSSG has a Steering Committee (SC) made of 9 members elected by the membership for a 3-year term, renewable once. In 2012, the IPMSSG held their first international meeting dedicated to clinical trials in pediatric MS and appointed a Clinical Trial Task Force (CTTF) to specifically address issues related to clinical trials in this age group.^30^ The CTTF is composed of 4 members, elected every 3 years by the IPMSSG SC. The CTTF has several core responsibilities, key among them the careful vetting of Pediatric Investigation Plans, which are now mandated by the FDA and the EMA for new agents developed in adults.

**Table 2:**
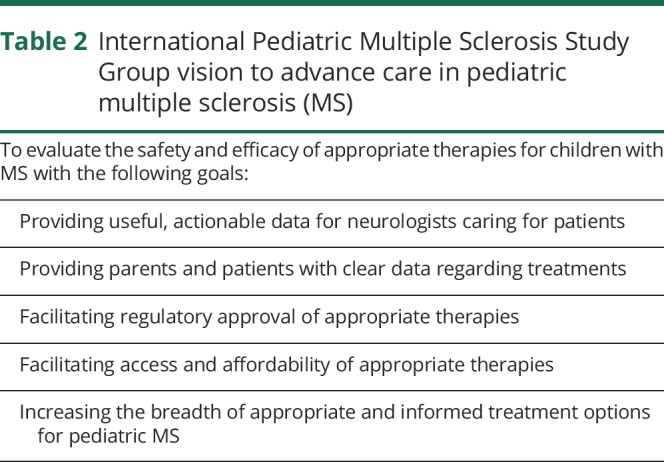
International Pediatric Multiple Sclerosis Study Group vision to advance care in pediatric multiple sclerosis (MS)

The IPMSSG SC and several national MS Societies recognized the need to convene a meeting to update the consensus for clinical trials in pediatric MS.^30^ This meeting was hosted by the Canadian and US MS societies, and included key stakeholders representing MS societies and patients' perspectives. In addition to the IPMSSG SC, an MS clinical trial statistician (M.-P.S.) and an ethicist (Dr. Alison Bateman-House) specialized in clinical trials were invited to join the meeting to share their expertise with the group. This group of 14 individuals met in New York City on January 18 and 19, 2018. A consensus was established that is outlined below, which was then vetted by IPMSSG members to ensure broad representation. Of the 162 members who were contacted, 70 from 23 countries provided feedback with only one disagreeing with some of the recommendation wording.

During the meeting, the group reviewed progress since the 2012 consensus paper,^30^ and identified areas of agreement to implement in future trials of new MS agents and areas that needed additional discussions. The literature concerning pediatric MS and use of MS therapeutic agents in pediatric MS was reviewed as well as new unpublished data pertaining to use of disease-modifying therapies (DMT) in various countries, and clinical trial design options with respective power. Feedback from physicians providing care for pediatric MS, most of whom had participated in clinical trials, was obtained in 2017 by the CTTF, and the perspectives from patients and their families regarding pediatric MS clinical trials were obtained through surveys performed in England and France in 2017.^31,32^

Table 3 provides the key recommendations of the IPMSSG SC, for which 100% agreement was reached.

**Table 3:**
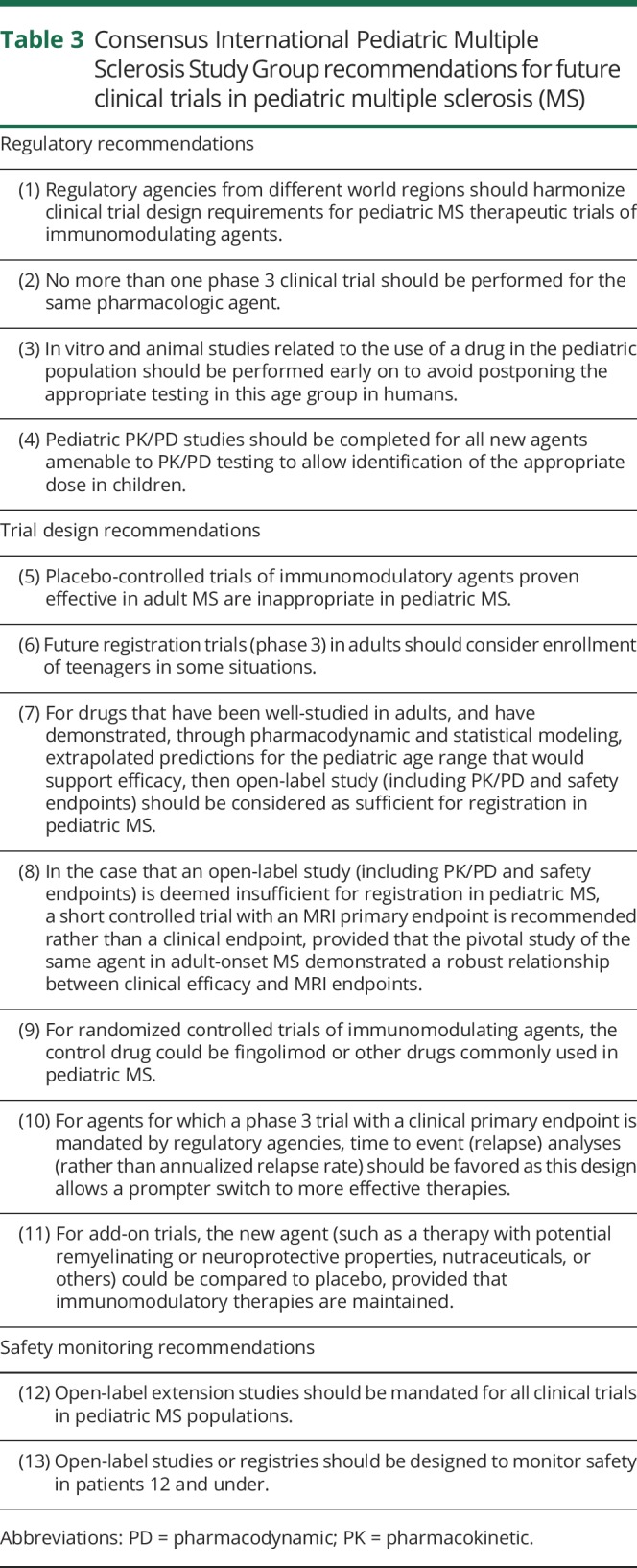
Consensus International Pediatric Multiple Sclerosis Study Group recommendations for future clinical trials in pediatric multiple sclerosis (MS)

## Status of clinical trials in pediatric MS

Three clinical trials of agents approved for the treatment of adult relapsing-remitting MS were completed in the pediatric population in 2014 and 2017 (table 1). The natalizumab pharmacokinetic/pharmacodynamic (PK/PD) study was a 16-week open-label study (NCT01884935) completed in 2014. FOCUS was a 6-month open-label study of dimethyl fumaric acid 240 mg twice a day in children 30 kg or more completed in 2016.^24^ This study is followed by a 2-year open-label extension. PARADIGMS was a phase 3 randomized controlled trial (RCT), with double dummy design comparing fingolimod vs weekly IM interferon-β-1a for up to 2 years.^25^ This study is followed by a 5-year open-label extension. Three trials are ongoing with teriflunomide, dimethyl fumaric acid, and alemtuzumab (table 1).

## Status of therapeutic access

Despite the paucity of clinical trial evidence, several MS therapies are available (sometimes approved) to treat pediatric MS depending on the country, occasionally with variability within countries based on provincial rules. Examples include interferon-β and glatiramer acetate in Italy, England, France (with special precaution under 12), the Netherlands, Argentina, Canada, and the United States. While natalizumab is available in France (>12 years of age), the Netherlands, England, Italy, Argentina, and Canada for patients with breakthrough disease on interferon or glatiramer acetate, it is available in the United States for most pediatric patients depending on health insurance restrictions and availability of infusion centers certified for drug administration. Teriflunomide is available in England, Canada, and the United States for pediatric MS. Fingolimod is now approved for children between 10 and 18 years in the United States, Europe, Argentina, and Canada. Over the last 6 years, the availability of some DMT for children and teenagers with MS and the recognition of an early clinical impact of the disease, especially on cognition, have prompted recommendations to initiate treatment shortly after diagnosis.^33^ These considerations, in addition to the regulatory approval of fingolimod in several countries as first-line therapy, affect the feasibility and ethics of future placebo or active comparator clinical trials in pediatric MS, including the choice of an active control.

## Challenges of pediatric MS clinical trials

Several challenges have been identified in the last 5 years through the conduct of clinical trials in the pediatric age group.

### Time to complete study enrollment

The first randomized active comparator phase 3 trial in pediatric MS (PARADIGMS) took 3 years (July 2013–August 2016) to enroll 215 participants at 80 centers in 25 countries to achieve enrolment. TERIKIDS is a phase 3 randomized placebo-controlled study that took 3.5 years (June 2015–January 2018) to enroll 166 patients among 77 sites in 22 countries. Enrollment times were substantially protracted relative to adult trials of the same agents. The reasons underpinning the slow enrollment in pediatric MS trials include (1) the limited pool of possible candidates, which necessitated addition of numerous study sites, leading to a prolonged launch period; (2) the limited prior clinical trial experience by participating sites hindered timely site preparedness; and (3) a low rate of consent from eligible participants and their families, who cited issues with the study design, time to travel to sites, and the burden of frequent study visits.^31^

### Risk of incomplete enrollment

The protracted timeline for clinical trials in pediatric MS may lead to incomplete enrollment.^32^ Incomplete trials (a.k.a. “ghost trials”) are inadequately powered to meet study endpoints, leaving enrolled participants and the entire pediatric MS community without the requisite data regarding safety and efficacy of the agent under study.^34^

### Limited pool of possible clinical trial candidates

In the United States, the prevalence of pediatric MS is estimated at less than 5,000 patients.^35,36^ This is in contrast with up to 800,000 American adults living with MS.^37^ While worldwide prevalence data are lacking, a conservative estimate suggests that there are fewer than 10,000 pediatric MS patients currently younger than age 18 years. As median age at pediatric MS onset is 15 years, patients remain in the pediatric age group for a limited time after diagnosis.^38^

### Site-specific challenges in pediatric MS trials

Many investigators who participated in the first pediatric MS trials had never participated in trials before. Finding appropriate research coordinator support for a study that would enroll very few patients at a given site, navigating regulatory issues, and planning for an appropriate budget were some of the challenges encountered.^31^ Furthermore, as exemplified by the PARADIGMS study, a pediatrician had to be identified for several visits to confirm Tanner stage and normal development, in addition to the treating and Expanded Disability Status Scale neurologists, and the physician overseeing the first-dose administration. As such, multiple providers were required for a very small number of enrolled participants.

### Financial risk

Most sites participating in the first pediatric MS trials enrolled between 0 and 2 patients. This means sites invested a considerable amount of time and energy upfront to complete regulatory work despite low expected enrollment, which was often not remunerated given low per-patient reimbursement. Coordinator support was particularly financially difficult as the number of planned participants was insufficient to justify full-time support yet many smaller sites do not have a pool of coordinators capable of providing part-time hours. Finally, low per-site enrollment also increases per-site costs for companies sponsoring the trials given their time investment in site launch and costs for monitors who visited sites for only a few participants.

### Patients and family endorsement

Prior to 2013, there had never been any clinical trials of agents for pediatric MS. As such, parents and patients had no prior exposure to clinical trial options and to the implications of participation. A survey conducted in England and France, and focus groups conducted in the United States with pediatric patients with MS and their parents, emphasized that it was challenging for families to take time off from work and school to participate in a trial.^32^ Children were often altruistic and eager to contribute to science. Parents expressed concerns about the safety of new drugs and the possibility to be randomized to placebo or a potentially less efficacious drug. Further compounding parental and patient decision-making is the off-label access to the medication under study in some countries. Finally, a double-dummy design did not resonate with some families, who disliked the idea of placebo injections. However, most families expressed interest in participating in future clinical trials.

## Consequences of the first completed phase 3 RCT in pediatric MS

PARADIGMS was the first RCT completed in pediatric MS.^25^ The study showed superiority of fingolimod to weekly IM interferon-β-1a treatment for the primary (annualized relapse rate) and secondary endpoints. Several consequences are expected from the trial results. First, the feasibility of conducting and completing MS trials in this age group was demonstrated, despite prolonged enrollment period. Second, demonstration of efficacy was achieved with a modest sample size (approximately 100 participants per study arm). Third, the trial results have led to the approval of fingolimod for patients with pediatric MS (age 10 years and older) in several countries. Of note, approval for use of fingolimod in pediatric MS occurred 8 years following the initial approval in adult MS.

While PARADIGMS provides the first level 1A evidence of effectiveness of a therapeutic agent in pediatric MS, other therapies, notably typical first-line treatments such as interferon-β and glatiramer acetate, remain appropriate for some patients and have over 15 years of safety data in that age group. Defining which pediatric patients with MS would be better served by fingolimod requires careful clinical and family dialogue. Finally, the higher efficacy of fingolimod compared to weekly interferon in the pediatric trial raises the question of what agent should be used as active comparators in future trials, which in turn will affect power considerations.

## Consensus on trial designs for pediatric MS

The SC reviewed information from other pediatric disorders for which agents are typically tested first in adults with the disease. The situation for antiepileptic agents was particularly informative.^39,40^ In the context of specific pediatric epilepsies, the FDA has accepted the extrapolation of efficacy to pediatrics based on adult trial data down to age 2 years, with a requirement for only PK and PD study.^39,40^ The decision to make this extrapolation rested on evidence that mechanisms and pathobiology was shared across the age span. The IPMSSG SC reviewed and endorsed the considerable evidence supporting the contention that MS is also an age span disease.^3–7^

The SC agreed that further studies of MS treatments are needed in pediatric MS to provide information, including dosing, on alternate drugs, especially for newer agents with different mechanisms of action and safety profiles. The SC agreed that trial design should be chosen according to the class of medication (i.e., anti-inflammatory vs neuroprotection vs restorative/remyelination).

Consensus recommendation for regulatory agencies: Pediatric PK/PD studies should be completed for all new agents amenable to PK/PD testing to allow identification of the appropriate dose in children.

## Ethical considerations

The principle of nonmaleficence in medical ethics requires that a procedure does not harm the patient involved or others. All members of the group emphasized ethical concerns about studies that included the use of placebo.^41,42^

Based on the evidence that RRMS in children and adolescents is associated with a higher relapse rate and more rapid accrual of new lesions relative to adult-onset disease and that highly active disease associates with a higher risk of future disability, the group proposed the following:

Consensus recommendation for trial design: Placebo-controlled trials of immunomodulatory agents proven effective in adult MS are inappropriate in pediatric MS.

Other approaches for studying medications that are already registered for adults but need further evaluation in the pediatric age group to address safety rather than focus on efficacy include (1) limiting investigation to PK/PD studies to inform dosing for the pediatric age group or (2) clinical trial designs with shorter duration (such as 6 months) that include MRI rather than clinical endpoints. For drugs not yet approved in adults, it was suggested to consider enrollment of those 12 years and older in future registration phase 3 trials of adult MS (see next section). In either scenario, it is critical to enroll pediatric patients with MS into open-label registries that monitor long-term safety of new agents including those under the age of 12.

## Inclusion of children in adult studies

The SC debated on the ethics and feasibility of enrolling teenagers in phase 3 trials performed in adults with MS to promote early access of younger patients to new treatments at the time of approval. The consensus was that this might be considered only for agents with strong safety data in adult MS phase 2 trials, strong safety data already established in phase 2 and 3 trials for conditions other than MS, for agents (such as vitamin D) for which safety data exist in other pediatric populations, and for agents with promising safety based on PK/PD studies in pediatric MS. It was considered reasonable to avoid exposure of young children such as those who are in the preadolescent age range to new agents under development for which safety was not yet demonstrated.

Consensus recommendation for clinical trial design: Future registration trials (phase 3) in adults should consider enrollment of teenagers in some situations.

## Clinical trial designs

The group agreed that trial design should be chosen according to the primary class of medication (i.e., anti-inflammatory vs neuroprotection vs restorative/remyelination).

### Anti-inflammatory agents

The SC discussed feasible trial designs to test anti-inflammatory agents already approved after phase 3 trials in adult MS. A fundamental consideration is to design trials that can feasibly enroll in a timeframe that advances access to effective therapies. Study designs requiring very large cohorts need to be avoided to prevent ghost trials.

Consensus recommendations for trial design:

- For drugs that have been well-studied in adults, and have demonstrated, through pharmacodynamic and statistical modeling, extrapolated predictions for the pediatric age range that would support efficacy, then open-label study (including PK/PD and safety endpoints) should be considered as sufficient for registration in pediatric MS. Data extrapolated from young adult datasets and Bayesian analysis may be used to augment efficacy data.^43^
- In the case that an open-label study (including PK/PD and safety endpoints) is deemed insufficient for registration in pediatric MS, a short controlled trial with an MRI primary endpoint is recommended rather than a clinical endpoint, provided that the pivotal study of the same agent in adult-onset MS demonstrated a robust relationship between clinical efficacy and MRI endpoints. Two independent meta-analyses confirmed that treatment effect on relapses can be precisely estimated by the effect observed on MRI lesions in adults for all the approved drugs, giving a solid base to this recommendation.^27,28^ The results of the second meta-analysis,^27^ validating the results of the previous one,^28^ give the rationale to regulatory agencies to accept MRI markers formally as surrogate outcomes in pediatric MS trials. Such trial designs will require fewer participants, given the greater treatment effect measured by MRI relative to clinical outcomes (such as relapse rate), and can be achieved using a shorter trial duration (typically 6 months), making these trials more feasible in children. Given that MRI as a primary outcome may not be permitted by regulatory agencies for all such trials, we recommend the following:

Consensus recommendation for trial design: For agents for which a phase 3 trial with a clinical primary endpoint is mandated by regulatory agencies, time to event (relapse) analyses (rather than annualized relapse rate) should be favored as this design allows a more rapid switch to therapies with superior efficacy.^44^

The SC discussed that ideally one study with multiple drug arms could be appealing. However, such studies would be challenging to implement as timelines to test agents may vary due to pediatric investigation plan pressures, and sequence of study visits and safety monitoring may vary depending on the agent. The funding mechanism of a trial evaluating drugs manufactured by different companies seemed to be an additional challenge difficult to address unless funding was channeled through research foundations or public research entities.

### Neuroprotection and remyelination

The potential for current therapies to enhance remyelination and neuronal/axonal repair remains unclear. As trials emerge exploring compounds with potential neuroprotective or neuroreparative mechanisms, such drugs are likely to be added to ongoing immunomodulatory treatments (at least in RR disease, which is the only form of MS seen in the pediatric population). When such treatments are available for study, the following recommendation was proposed:

Consensus recommendation for trial design: For add-on trials, the new agent (such as a therapy with potential remyelinating or neuroprotective properties, nutraceuticals, or others) could be compared to placebo, provided that immunomodulatory therapies are maintained.

## Regulatory issues

### Harmonization of regulatory guidelines

Over the last 6 years, the SC has been concerned with the heterogeneity of FDA and EMA requirements for clinical trial design for pediatric MS. Examples of study designs utilized in pediatric MS include a double-dummy active control design for PARADIGMS, a placebo-controlled design for TERIKIDS, an open-label randomized trial with active control for CONNECT, and open-label observational study for LEMKIDS (table 1). This lack of harmonization has led to highly diverse designs for the ongoing trials, inherently limiting ability to compare clinical trials to one another. The imperative to gain consistent clinical outcome data from therapeutic trials leads to the following:

Consensus recommendation for regulatory agencies: Regulatory agencies from different world regions should harmonize clinical trial design requirements for pediatric MS therapeutic trials of immunomodulating agents.

Furthermore, differing opinions between regulatory agencies has also led to different clinical trial designs for the same drug in different world regions, as in the case of dimethyl fumarate, where at least 3 studies have been launched. Given the limited number of pediatric patients with MS, having competing trials, particularly for single agent, has a negative effect on the ability to fully recruit and runs the risk of ghost trials. The SC hopes to work with the EMA and FDA so harmonized recommendations can be used for the design of future clinical trials in pediatric MS, and strongly endorses the following:

Consensus recommendation for regulatory agencies: No more than one phase 3 clinical trial should be performed for the same pharmacologic agent. Harmonizing regulatory requirements from different world regions is required to ensure a single study per new agent if the study enrolls across multiple countries.

### Pediatric in vitro and in vivo models

The FDA and EMA should consider not only data from adult MS studies, but also preclinical data informing on risks relative to the pediatric populations. If such studies are requested only after completion of the phase 3 studies in adults, then the design and launch of pivotal trials in pediatric MS are delayed.

Consensus recommendation for regulatory agencies: In vitro and animal studies related to the use of a drug in the pediatric population should be performed early on to avoid postponing the appropriate testing in this age group in humans.

### Reporting of trial results

The SC also agreed that any results from trials performed in pediatric MS participants should be presented and made available to the public within 12 months of result analyses as requested by the NIH (clinicaltrials.gov), so treatment decisions could be implemented timely in young patients.

## Strategies to enhance patient participation in trials

Based on the experience of the SC and broader feedback coming from a survey of the IPMSSG membership, it was recommended that several approaches should be considered to ease participation in pediatric MS trials considering the substantial time commitment for children (i.e., school) and parents (i.e., work).^31^ Trial designs should limit the number of in-person visits and consider use of telephone or virtual telehealth visits when feasible for safety monitoring visits. Scheduling of such visits could also occur during after school hours or on weekends to reduce school absenteeism. Outcome metrics that engage the pediatric population, such as symptom tracking via applications downloaded onto mobile devices, are future considerations. The use of a local laboratory for safety biological tests or a visiting nurse would be additional steps towards decreasing the burden for families participating in trials. Provision of parental compensation to cover time lost at work when bringing their child for study visits or costs associated with care of young siblings should be considered in study budgets. Finally, study visits should be shortened and efficiency of the visit maximized.

To that end, it is recommended that a short cognitive screening approach with only one or just a few tests be included. This differs from the prior recommendation in 2013 since that proposed battery was 45 minutes or more in length and difficult to implement. Brief cognitive screening approaches that have been successfully studied in pediatric MS include a pediatric version of the 3-test Brief International Cognition Assessment in MS (BICAMS,^45^ the Symbol Digit Modality Test [SDMT],^46^ a computer administered version of the SDMT,^47^ or other brief sensitive computer-administered measures).^45^

While the value of MRI as either a primary or secondary outcome is endorsed, the frequency of imaging should be carefully considered. For example, in 2-year trials, annual brain MRI scans were believed appropriate as this frequency would match clinical practice. The group also agreed that administration of IV contrast products were only necessary at baseline to better define disease activity, but was not necessary for subsequent MRI scans as the number of new or enlarging T2-bright foci was a more reliable endpoint than contrast-enhancing lesions in studies with infrequent scans. Limiting the use of contrast agents addressed both a parental concern regarding the possible long-term toxicity of repeated administration of these agents^48^ and also shorter duration of study visits.

Long-term safety data are arguably one of the most important facets required to advance the care of pediatric MS patients, and to ensure that treatment during childhood and adolescence does not expose patients to future risk. Given this, the SC strongly endorses the following:

Consensus recommendations for safety monitoring: Open-label extension studies should be mandated for all clinical trials in pediatric MS populations. Given that monitoring participants as they transitioned to college, sometimes moving far away from home, the use of novel participant interaction methods, such as mobile device patient-entered data, could help maximize retention in this critical phase of safety monitoring.

The SC recognized that most trials did not enroll pediatric patients with MS under the age of 10, and that those enrolled in the 10–12 years age category were very few, to some extent limiting safety information for new drugs in the very young. It was recognized that this age group is very small (i.e., represents 20% or so of all pediatric patients with MS), and that their participation in trials is more challenging than teenagers, as, for example, brain MRI scans may require sedation in the very youngest children. Given that no clinical trial is likely to be powered to measure efficacy in this age group, the SC proposes the following:

Consensus recommendations for safety monitoring: Open-label studies or registries should be designed to monitor safety in patients 12 and under.

## Future directions

The SC was pleased to see the overall progress regarding care, access to DMT, and testing of new agents for pediatric MS in the last 6 years but acknowledged several critical areas that should be addressed by future research in that age group. Notably, priorities should include the validation of telehealth/remote visits, the development of clinical trial designs based on MRI variables as a surrogate endpoint, and the harmonization of regulatory requirements for testing of new treatments in pediatric MS. Specific inclusion criteria should also be refined for enrollment in pediatric MS clinical trials in the future such as serostatus for aquaporin-4 and myelin oligodendrocyte glycoprotein immunoglobulin G. Future work will determine the role of brain volume, optical coherence tomography, and serum levels of neurofilament as possible outcome measures of interest for clinical trials.

## Glossary

CTTF: Clinical Trial Task Force
DMT: disease-modifying therapies
EMA: European Medicines Agency
FDA: Food and Drug Administration
IPMSSG: International Pediatric Multiple Sclerosis Study Group
MS: multiple sclerosis
PD: pharmacodynamic
PK: pharmacokinetic
RCT: randomized controlled trial
RR: relapsing-remitting
SC: Steering Committee
SDMT: Symbol Digit Modalities Test
